# Radiology Training in Emergency Medicine Residency as a Predictor of Confidence in an Attending

**DOI:** 10.7759/cureus.6615

**Published:** 2020-01-09

**Authors:** Eric Blazar, Danial Mitchell, Jason D Townzen

**Affiliations:** 1 Emergency Medicine, Inspira Medical Center, Vineland, USA

**Keywords:** emergency medicine, radiology, plain film, resident training

## Abstract

Introduction: At present, there exists no standardized curriculum for the interpretation of plain film radiography for emergency medicine (EM) training programs that have been adopted by an accrediting body. Education geared towards plain film interpretation is highly variable and institutionally specific. This highly variable education is dependent upon institutional resources, availability of real-time radiology interpretations, formalized radiology instruction, in addition to self-directed study. Furthermore, it is unclear whether the presence of a radiology residency program at the same institution will positively or negatively impact the radiographic education of the EM resident. In a community practice setting, EM providers may encounter several scenarios in which they must rely on their own independent interpretations during radiology coverage gaps. The goal of this study was to assess whether the amount of formal radiology training correlates with the confidence in the interpretation of radiographs following residency graduation early in a junior attending’s career.

Methods: A survey study with 14 questions was distributed to EM attendings utilizing social media. Over a two-month period, 218 responses were obtained and statistical analysis was performed utilizing a chi-square test. Three survey questions with multi-variable answers were compressed into two variables for statistical analysis.

Results: Only 30% of survey participants indicated universal radiology coverage; 30% also responded that they did not feel prepared to interpret plain film radiographs upon residency completion. There were four statistically significant factors associated with higher confidence in interpreting radiographs upon residency graduation. Physicians were more likely to feel confident in reading radiographs if they (1) graduated from a program with no radiology residency present, (2) if their residency was located in a non-tertiary training facility, (3) if most of their radiograph learning occurred on shift and (4) if they made clinical decisions based on their own interpretations frequently. 40% of physicians reported they were more confident currently in interpreting radiographs than when they first completed residency.

Conclusion: Steps should be taken to ensure that graduating residents are being prepared to interpret plain film radiographs as many providers will be required to do so independently in future practice. Emphasis should be focused towards on-shift teaching of these skills. Graduates at greatest risk of lower confidence train at large tertiary care centers with concomitant training of radiology residents. By emphasizing on off-shift strategies for the interpretation of plain film radiographs, residents will build confidence and develop the ability to perform these necessary skills early in one's career.

## Introduction

Currently, the Accreditation Council for Graduate Medical Education (ACGME) does not specify a minimum amount of radiology training that is necessary for the graduation of emergency medicine (EM) residents [1]. Variables that influence radiology training for EM residents include, but are not limited to, the amount of formal lecture time dedicated to reading radiographs, radiology support that is employed by the hospital (24/7 radiograph reading vs. only during peak hours), and the need to make clinical decisions without official radiology interpretations of radiographs. A recent 2018 study from the journal Academic Radiology found that the radiology interpretation skills of incoming interns are consistently below the expectations of program directors in multiple specialties [2]. While emergency medicine training in radiograph interpretation utilizes both real-time on-shift instruction as well as didactic lecturing, the actual amount of formalized teaching is highly variable when comparing different programs.

Multiple studies addressing radiological training in surgical residency have been performed. A 2016 study from the American Journal of Surgery reported that over 90% of surgical attendings and over 85% of surgical residents made clinical decisions in the acute setting without an official read from a radiologist [3]. In this same study, both faculty and residents were asked if they thought a formal curriculum was needed to aid in their interpretation of radiographic studies. 69% of surgical attendings and 74% surgical residents positively responded. EM residencies surveyed in 2002 reported over 60% of sites used independent interpretation of radiographs for clinical decision-making. This percentage reached almost 80% on the weekends [4]. While no similar study exists in EM assessing the thoughts of program directors and residents on the need for formal EM residency radiology curricula, the similarities between general surgery and EM on use of radiographs for key clinical decisions suggests a possible need for a more structured approach to radiographic training in EM residency.

Multiple studies have assessed the clinical accuracy of EM attendings’ interpretation of plain radiographs. Opinions and results are mixed, and while some studies have found that the rate of misdiagnosis is low, with the overall discrepancy rate reported at around 1%, other studies have reported higher rates of discrepancy even among common films such as a chest radiography [5-6]. However, studies done specifically with residents as the interpreters suggest a far higher miss rate, even among senior residents [7]. This suggests that radiology learning continues and markedly improves into early attending-hood. Recent graduates will be forced to make clinical decisions with the knowledge they gained in residency, but hold significantly lessened expertise in comparison to their older colleagues. A 1998 study in Annals of Emergency Medicine showed that discrepancies between radiologists and emergency physicians were lower when the EM physician was confident in their read [8]. Options for improving radiology education in EM residents include formal lectures by radiologists, dedicated radiology rotations, and formal review of discrepancies [9-11].

While many EM residency graduates train in large academic centers with many resources, most jobs in EM are outside of these academic centers. EM providers most likely do not have radiologists actively reading at all hours in these community settings. As an attending, many graduates will then be required to make decisions based on radiographs from their own interpretations without official reads from a radiologist.

The goal of this study was to assess whether the amount of formal radiology training received during EM residency is correlated with the confidence of attendings in reading radiographs in the emergency department. We attempted to discern the amount and type of formal radiology training currently being performed in EM residencies throughout the United States. We also studied the current level of radiological support from attending radiologists at respondent’s hospitals. We aimed to survey the comfort level of newer attending physicians (within five years of graduation) reading radiographs and making clinical decisions without official radiologist interpretations. We then attempted to correlate this comfort in new attendings with the amount of training that the physician received in radiography interpretation during their residency training. 

## Materials and methods

A 14-question survey was prepared, with the intention of distributing it online to EM attendings, in order to provide answers regarding their training as a resident, as well as to evaluate their confidence in reading plain film radiographs (Table 1).

**Table 1:** Physician Survey

Questions	Answer Choices
1. Did you graduate from a US accredited ACGME/AOA program?	A. Yes B. No
2. How would you describe the primary hospital that you worked at during residency?	A. Large academic tertiary care center with a significant number of residencies, and sub-specialty fellowship programs available
	B. Non-tertiary hospital, with basic residency programs, limited to no fellowship/sub-specialty training
	C. Community hospital with few residency programs
Where was your residency located?	A. Northeast (PA, NJ, NY, CT, RI, MA, VT, NH, ME, DE, MD)
	B. South ( DC, VA, WV, NC, SC, TN, GA, AL, MS, FL, AK)
	C. Midwest (KY, ND, SD, NE, KS, MN, IA, MO, WI, MI, IL, IN, OH)
	D. West ( CA, ID, NV, MT, WY, UT, CO, AZ, NM) or Pacific (WA, OR, HI)
4. Was there an accredited Radiology residency at the program that you trained at?	A. Yes B. No
5. How many years of experience do you have as an Emergency Medicine attending?	
6. During your residency training, where did you feel most of your learning about interpreting X-rays occur?	A. On shift
	B. During conference/didactics (formal lectures)
	C. Reading/Self-study
7. During your residency training, which resource did you find the most helpful to your learning of interpretation of radiographs?	A. Teaching on shift
	B. Formal lectures in conference/didactics
	C. Self-study with books/board review materials
	D. Self-study with YouTube or other digital media
8. On a scale of 1-10, with 10 being very frequent (at least monthly), 5 being Bi-monthly, and 1 being rarely, how would you rate the frequency of formal medical education (lectures) that you received on interpreting X-rays during residency?	
9. At the main site of your residency training, how often did you make clinical decisions/interpret radiographs without an official interpretation by a radiologist?	A. Never (All clinical decisions come after official radiology reads)
	B. Rarely (At least once per month)
	C. Occasionally (At least once per week)
	D. Often (most shifts)
10. At your first attending position out of residency, what level of radiology support was there for radiograph reading?	A. 24-hour coverage by radiologist in house for X-rays
	B. Radiologist reads X-rays during the day. EM physician responsible for nights and weekends
	C. Minimal reads available in real time. EM physicians responsible for reads the majority of the time
11. Please rate your confidence currently reading and interpreting radiographs, and subsequently making clinical decisions based on your reads without an official read by a radiologist?	A. Extremely confident: Rarely request overreads/clarification from radiologist. Frequently make confident medical decisions based on your own interpretation.
	B. Mostly confident: occasionally uncomfortable/unsure and will occasionally (Weekly to monthly) request overread from radiologist prior to making medical decision. Mostly makes medical decisions based on own reads.
	C. Somewhat confident: Will request radiology overread/clarification approximately weekly. Confident in diagnosing major pathology but can miss minor details or rarer pathology.
	D. Unconfident: Often (Most shifts) requesting overreads/clarification by radiologist prior to making medical decisions. Will order CT scans more frequently if unsure of X-ray diagnosis.
12. Please rate your confidence right out of residency reading and interpreting radiographs, and subsequently making clinical decisions based on your reads without an official read by a radiologist?	A. Extremely confident: Rarely request overreads/clarification from radiologist. Frequently make confident medical decisions based on your own interpretation.
	B. Mostly confident: occasionally uncomfortable/unsure and will occasionally (Weekly to monthly) request overread from radiologist prior to making medical decision. Mostly makes medical decisions based on own reads. makes medical decisions based on own reads.
	C. Somewhat confident: Will request radiology overread/clarification approximately weekly. Confident in diagnosing major pathology but can miss minor details or rarer pathology.
	D. Unconfident: Often (Most shifts) requesting overreads/clarification by radiologist prior to making medical decisions. Will order CT scans more frequently if unsure of X-ray diagnosis
13. Do you wish you had more didactic time/lectures on X-ray interpretation during your residency?	A. Yes B. No
14. Overall did you feel adequately prepared after residency to interpret X-rays and make clinical decisions from your interpretations without an official radiologist read?	A. Yes B. No

Participation in this survey was voluntary and anonymous. The study was piloted to four Inspira Medical Center EM attendings for errors and/or language misinterpretation. These answers did not factor into the results, as Inspira EM physicians were not eligible for this study. The IRB at Inspira Medical Center reviewed the study protocol and methods, and approved the study for data collection; it was exempt for ongoing review.

From November of 2018 to December of 2018, the 14-question survey was posted to the emDOCS Facebook group, which comprises a nationwide sample of EM physicians at all levels of experience. The survey was posted to the group a total of three times and 218 responses were obtained in total. Responses were downloaded into an anonymous excel spreadsheet for data analysis. Response percentage and frequency were gathered to analyze the responses. A chi-squared test was utilized to examine the data. A t-test was used to examine the difference between questions with two groups and continuous variables. Three questions (2, 6 and 7), which originally were written with multiple answer choices were compressed into two choices in order to obtain statistical significance. Table 1 shows the questions and answer choices, with the collapsed answers bolded.

## Results

The primary objective of this study was to determine what factors in residency training correlated with confidence in interpreting radiographs as an attending. All 218 respondents stated they graduated from the American Osteopathic Association (AOA) or ACGME-accredited residencies in the US. Location of residency training was dispersed across the country, with no region being significantly over-represented. 30% of respondents in this survey answered they did not feel prepared to read plain film radiographs upon residency graduation. Statistical analysis showed there were four statistically significant (p value <0.05) factors associated with higher confidence in interpreting radiographs as a junior attending. Physicians were more likely to feel confident in reading radiographs if they graduated from a program with no radiology residency present, if their residency was located in a non-tertiary training facility, if most of their radiograph learning occurred on shift, and if they made clinical decisions based on their own reads frequently. Table 2 summarizes these results.

**Table 2:** Survey Analysis

Statistically significant (p<0.05) factors associated with residents feeling adequately prepared			
		Feel Adequately Prepared	
Question	Response	No	Yes
Residency Hospital Type **Collapsed to tertiary vs. non-tertiary**	Community hospital, few residency programs	3 (12.00%)	22 (88.00%)
	Large academic tertiary care center, significant number of residencies	62 (38.27%)	100 (61.73%)
	Non-tertiary hospital, limited to no fellowship/sub-specialty training	4 (13.79%)	25 (86.21%)
Accredited Radiology	No	12 (18.75%)	52 (81.25%)
	Yes	57 (37.50%)	95 (62.50%)
Learned Interpreting X-rays **Collapsed to on-shift vs. off-shift**	During conference/didactics	10 (50.00%)	10 (50.00%)
	On shift	49 (27.84%)	127 (72.16%)
	Reading/Self-study	10 (62.50%)	6 (37.50%)
Most Helpful Resource **Collapsed to on-shift vs. off-shift**	Formal lectures in conference/didactics	9 (50.00%)	9 (50.00%)
	Self-study with YouTube or other digital media	7 (63.64%)	4 (36.36%)
	Self-study with books/board review materials	10 (38.46%)	16 (61.54%)
	Teaching on shift	43 (27.39%)	114 (72.61%)
Interpret Radiographs W/O official reads during residency	Never (clinical decisions after official radiology)	14 (58.33%)	10 (41.67%)
	Occasionally (At least once per week)	20 (40.82%)	29 (59.18%)
	Often (most shifts)	18 (18.18%)	81 (81.82%)
	Rarely (At least once per month)	17 (38.64%)	27 (61.36%)

75% of the survey respondents indicated they trained in a large academic tertiary care center. Of the respondents who trained in a tertiary care center, only 60% felt adequately prepared to read radiographs on their own, which was significantly lower than the over 85% of respondents who trained at smaller hospitals reported. Similar numbers were also noted for respondents who trained at programs without co-existing radiology residencies. The newly graduated EM attendings that trained at institutions without a radiology program were significantly more confident with interpreting radiographs on their own.

Our survey noted that only 30% of graduating residents had 24-hour radiology support/reading available at their first job outside of residency, with most replies stating radiology was available during the day, but the EM physicians primarily performed radiograph interpretation on nights, weekends, and holidays.

Our initial survey included many options for education in radiograph interpretation. These options were simplified to teaching on shift vs. learning off shift in an effort to discern statistical significance. Analysis of our data showed teaching on shift correlated with increased confidence in reading X-rays. 72% of the respondents who reported the majority of their learning occurred on shift reported they were comfortable reading radiographs immediately following residency, which was significant when compared to off-shift learning (p<0.05). Almost identical numbers were found when respondents were asked the most effective learning strategy. Again over 70% of physicians were confident reading radiographs immediately following residency with on-shift learning as their primary source of education (p<0.05).

Physicians who made clinical decisions from their own interpretations frequently were more comfortable when they ultimately graduated from residency (p<0.05) and worked as a junior attending. Of the respondents who felt comfortable reading X-rays out of residency, over 80% stated they read radiographs frequently, at least once per shift, during residency. Contrarily, 11% (25) of total respondents reported they did not make medical decisions until a radiologist performed an official radiology read, and 60% (16) out of those 25 physicians felt uncomfortable reading radiographs and making clinical decisions from their interpretations.

**Figure 1:**
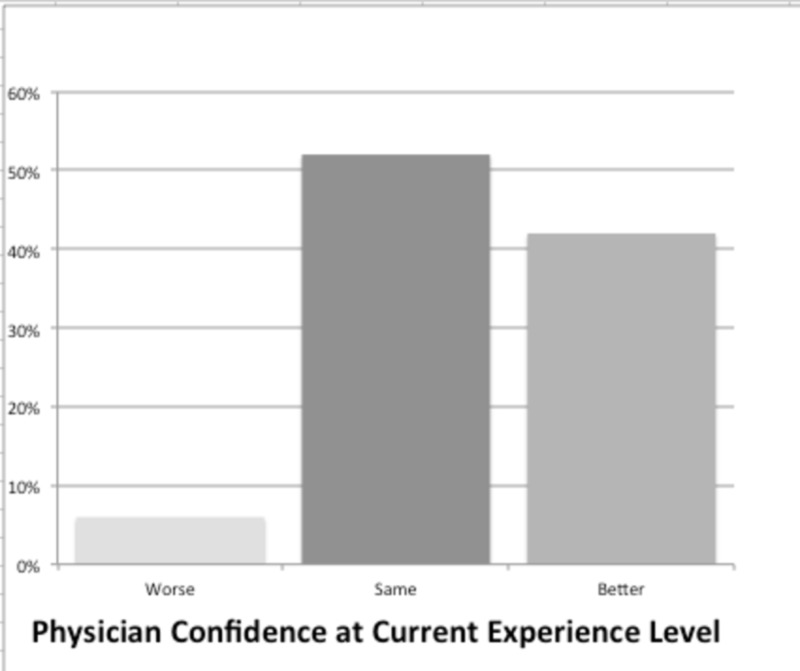
Physician vs New Graduate Confidence at Plain Film Interpretation

Physicians were also polled on their current confidence levels reading radiographs and their confidence levels right out of residency. Experience as an attending does seem to increase levels of confidence in reading radiographs. Answers to confidence level as a new attending and at their current level of practice were compared. 40% of physicians reported they were more confident currently in interpreting radiographs then when they first completed residency (Figure 1).

## Discussion

Interpreting radiographs is a necessary skill for EM physicians with respect to accuracy in diagnosis and proficiency/time management. A larger than expected percentage of physicians polled did not feel adequately prepared to interpret radiographs independently when they completed residency. It is common for a number of these graduating physicians to take positions at hospitals without 24-hour radiology support. The primary objective of this survey-based study was to identify factors in residency education that are associated with high confidence radiology interpretation for new EM attendings Training at a large tertiary care center and the presence of a co-existing radiology residency is correlated with decreased confidence. Multiple factors were found to be associated with increased confidence which included training at a non-tertiary hospital, training at a hospital without a coexisting radiology residency, teaching on shift as the primary method of learning radiograph interpretation during residency, and frequently interpreting radiographs on shift and making clinical decisions based on those interpretations.

Practice environments where EM residents train clearly have a significant impact on practice patterns and confidence as attendings. The majority of survey respondents trained in large tertiary centers, which generally have greater radiology support that likely results in EM residents reading their own studies less frequently and relying more on the official interpretation from the radiologist, who is always reading. With faster radiology study turnaround times, theoretically, residents may make less clinical decisions based on their own reads, which is likely indirectly impacting their education. 

Limitations

Our study was limited as it was a survey posted on a social network for a variety of physicians at varying stages in their practice career. Both training at a tertiary care center and having a co-existing radiology training program were statistically significant to our primary outcome measured, as well as being statistically significant in occurring together; it is difficult to interpret them independently. The confounding of these two factors is a limitation of our study. Further data studies would need to be done to possibly separate these cofounding factors. Also, due to our response numbers, multiple-survey questions were combined to binary options and thus, the best off-shift resource could not be discerned.

## Conclusions

EM residency programs must train their residents to be the most prepared for independent practice. Many graduates will not have radiology support and must be confident in making decisions based on their own radiograph interpretations. By continually teaching on shift, and encouraging residents to make reads in real time without an official radiologist interpretation, faculty teachers can optimize on-shift teaching. Other resources such as books, self-study and dedicated lecture time should be used as supplementary education, but these resources should not comprise the majority of an EM residents’ radiology education. One exception to this may be a dedicated radiology rotation, in which real-time feedback from a radiologist, in addition to consistent practice over a few weeks, could significantly improve a resident’s ability to read radiographs confidently in the ER. However, further study would have to be done to evaluate its effectiveness. Overall, as a result of our survey, we recommend residency leadership to specifically address and emphasize the importance of on-shift teaching of plain film interpretation as well as decision making from these interpretations. While supplemental teaching through lecture and self study is likely the easier method to teach plain film interpretation, it is clearly not as effective as strong on-shift teaching.
